# Avoidance as expectancy in rats: sex and strain differences in acquisition

**DOI:** 10.3389/fnbeh.2014.00334

**Published:** 2014-10-06

**Authors:** Pelin Avcu, Xilu Jiao, Catherine E. Myers, Kevin D. Beck, Kevin C. H. Pang, Richard J. Servatius

**Affiliations:** ^1^Graduate School of Biomedical Sciences, New Jersey Medical School, Rutgers Biomedical and Health SciencesNewark, NJ, USA; ^2^Stress and Motivated Behavior Institute, New Jersey Medical School, Rutgers Biomedical and Health SciencesNewark, NJ, USA; ^3^Department of Neurology and Neurosciences, New Jersey Medical School, Rutgers Biomedical and Health SciencesNewark, NJ, USA; ^4^Neurobehavioral Research Lab, Department of Veteran Affairs Medical Center – New Jersey Health Care SystemEast Orange, NJ, USA

**Keywords:** lever-press avoidance, anxiety, vulnerability, behavioral inhibition, Wistar-Kyoto (WKY) rat, expectancy, shock avoidance

## Abstract

Avoidance is a core feature of anxiety disorders and factors which increase avoidance expression or its resistance represent a source of vulnerability for anxiety disorders. Outbred female Sprague Dawley (SD) rats and inbred male and female Wistar-Kyoto (WKY) rats expressing behaviorally inhibited (BI) temperament learn avoidance faster than male SD rats. The training protocol used in these studies had a longstanding interpretive flaw: a lever-press had two outcomes, termination of the warning signal (WS) and prevention of foot shock. To disambiguate between these two explanations, we conducted an experiment in which: (a) a lever-press terminated the WS and prevented shock, and (b) a lever-press only prevented shock, but did not influence the duration of the WS. Thus, a 2 × 2 × 2 (Strain × Sex × Training) design was employed to assess the degree to which the response contingency of the WS termination influenced acquisition. Male and female SD and WKY rats were matched on acoustic startle reactivity within strain and sex and randomly assigned to the training procedures. In addition, we assessed whether the degree of avoidance acquisition affected estrus cycling in female rats. Consistent with earlier work, avoidance performance of female rats was generally superior to males and WKY rats were superior to SD rats. Moreover, female SD and male WKY rats were roughly equivalent. Female sex and BI temperament were confirmed as vulnerability factors in faster acquisition of avoidance behavior. Avoidance acquisition disrupted estrus cycling with female WKY rats recovering faster than female SD rats. Although termination of the WS appears to be reinforcing, male and female WKY rats still achieved a high degree (greater than 80% asymptotic performance) of avoidance in the absence of the WS termination contingency. Such disambiguation will facilitate determination of the neurobiological basis for avoidance learning and its extinction.

## Introduction

Avoidance in its various forms (experiential, emotional, and cognitive) is a common feature of all anxiety disorders (American Psychiatric Association, 2000). Development of anxiety disorders can be best explained by diathesis models, accounting for a complex interaction of individual vulnerabilities with environmental risk factors. A greater focus on individual differences in avoidance learning theories has the promise of providing insights into the epidemiology and course of anxiety disorders.

A number of organizing principles have been offered to understand acquisition, maintenance, and extinction of avoidance: those focused on associative learning of signs and signals (Seligman and Johnston, 1973; Lovibond, 2006), those focused on reinforcement (Mowrer, 1956; Bersh and Alloy, 1978; Hineline, 2001), and those using a cognitive framework (De Houwer et al., 2005; Declercq and DeHouwer, 2008; Dymond and Roche, 2009; Mitchell et al., 2009). Avoidance learning is most efficient when the subject's response terminates the WS and prevents the shock (shock avoidance). However, concurrent WS termination and shock prevention does not allow a clear interpretation of increases in responses. Is the animal responding to terminate the WS or is it responding to avoid shock? Classic work attempted to separate the reinforcing effects of WS termination and shock avoidance on avoidance learning (Sidman, 1955; Kamin, 1956; Sidman and Boren, 1957; Keehn, 1959; Lockard, 1963; Bower et al., 1965; Bolles et al., 1966; Owen et al., 1977). If WS termination was the primary reinforcement, then responses were controlled by the present, not the future. Converging evidence documented that an inability to terminate the WS merely affected the acquisition of avoidance response (Sidman, 1955; Kamin, 1956; Sidman and Boren, 1957; Keehn, 1959; Lockard, 1963; Bower et al., 1965; Bolles et al., 1966).

These previous studies examined avoidance learning in procedures that utilized shuttling, wheel running, and jumping as responses. These responses are all species specific defense reactions (SSDR), and therefore, the learning of the avoidance response has been previously questioned (Bolles, 1970). A specific case of avoidance is signaled lever-press avoidance; that is, a WS contingent with shock presentation provides the opportunity to learn that shock may be terminated (escape) or prevented (avoidance). Unlike SSDRs, arbitrary avoidance responses, like lever-press, are slowly and incrementally acquired. Through autoshaping, lever-presses are reinforced for shock termination and prevention.

Classic literature concentrates on explanations for normal avoidance acquisition, expression, and extinction in outbred strains. Yet, it is well-documented that abnormal avoidance expressions form the core of all anxiety disorders. Normal acquisition may only partially, and therefore incompletely inform the acquisition and maintenance of avoidance in anxiety disorders. A greater susceptibility to acquire pathological avoidance symptoms may cause some individuals to be more vulnerable to develop anxiety disorders. Factors that accelerate acquisition and promote perseveration of avoidance represent a diathesis for anxiety disorders—a learning-diathesis model. Accordingly, BI temperament (Rosenbaum et al., 1991, 1993; Schneier, 2003; Fox et al., 2005) and female sex (Wittchen and Hoyer, 2001; Vogt et al., 2005; Bleich et al., 2006; Foa et al., 2006; Hapke et al., 2006; Karamustafalioglu et al., 2006; Smith et al., 2008) are identified as independent vulnerability factors for the development of anxiety disorders.

As one moves toward understanding vulnerability factors in rapid avoidance acquisition and its persistence in animal models, a clear interpretation of the source of sensitivity is essential. Similar to human literature, female sex is also associated with greater risk for anxiety disorders in animal models. Female rats acquire lever-press avoidance faster than male rats (Heinsbroek et al., 1983; Beck et al., 2010). Resembling humans expressing BI temperament, inbred WKY rats display extreme withdrawal in the face of novel social challenges (Ferguson and Cada, 2004; Braw et al., 2006) and non-social challenges (McCarty et al., 1984; Pare and Schimmel, 1986; Pare, 1989; McAuley et al., 2009); and greater sensory motor reactivity compared to SD rats (Servatius et al., 1998; Beck et al., 2010). Acquisition of lever press avoidance is faster and expressed to a greater degree in WKY rats than SD rats (Servatius et al., 2008; Beck et al., 2010; Jiao et al., 2011; Perrotti et al., 2013). Furthermore, both females (Shors et al., 1986, 1998; Wood and Shors, 1998; Beck et al., 2008, 2012), and WKY rats (Ricart et al., 2011a,b) exhibit faster associative learning evident in eyeblink conditioning. Thus, enhanced avoidance acquisition may be through enhanced sensitivity to WS termination (driven by WS-shock associations) or prevention of shock. The existing data—in which WS co-terminated with the shock—fail to disambiguate these two possibilities.

The present study compared avoidance acquisition under two training procedures: (1) a lever-press terminated the WS and prevented shock occurrence (contingent WS) and (2) a lever press only prevented shock occurrence (non-contingent WS). Considering the interpretation of early findings (Sidman, 1955; Kamin, 1956; Sidman and Boren, 1957; Keehn, 1959; Lockard, 1963; Bower et al., 1965; Bolles et al., 1966), we expected acquisition of avoidance to be evident in the absence of the WS termination contingency, albeit slower than with a WS termination contingency. Further, we expected the female rats to express avoidance acquisition to a greater degree than male rats regardless of the training parameters. Similarly, we expected WKY rats to express avoidance to a higher degree than SD rats. Moreover, we expected an interaction of female sex and BI temperament such that female WKY rats would express avoidance to the highest degree of those tested. Thus, the vulnerability factors examined will demonstrate a specific enhanced sensitivity to the avoidance of impending shock, as opposed to momentary responses to the presence of the WS.

Additionally, we investigated the effect of avoidance acquisition on estrous cycle in female SD and WKY rats. Typically, physiological stress levels are maximal early in training which is commensurate with a relatively high degree of shock exposure. As avoidance is acquired, stress levels are reduced (Coover et al., 1973; Berger et al., 1981). Exposure to stress has been shown to have adverse effects on several aspect of the reproductive system (e.g., estrous cycle) in both humans (Genazzani et al., 1991) and animals (Gonzalez et al., 1994; Norman et al., 1994). Thus, it is expected that a disruption of the estrous cycle would be observed early during initial sessions of avoidance training but return during the later sessions when avoidance is learned and stress has presumably been reduced. With regard to WKY rats, the disrupted estrous cycle may recover faster than SD rats because WKY rats learn avoidance faster and to a greater extent than SD rats. On the other hand, if high asymptotic levels of avoidance is pathological, female WKY rats may continue to show irregular cycling throughout avoidance training.

## Materials and methods

### Animals

Forty-nine SD and forty-six WKY male (*n* = 48) and female (*n* = 47) rats (300–350 g, 8–10 weeks old; Harlan Sprague-Dawley Laboratories, Indianapolis, IN) were single-housed and kept on a 12:12 h light cycle (lights on 0700) with access to laboratory chow and water *ad libitum*. Upon arrival, rats were acclimated to the housing conditions for at least 2 weeks prior to experimentation. All experiments occurred between 0700 and 1200 h, in the light portion of the cycle. All studies were approved by the Institutional Animal Care and Use Committee (IACUC) in accordance with AAALAC standards.

### Open-field test

Naive rats were evaluated for locomotor activity in the open-field test, consistent with previous work (Servatius et al., 1995). The apparatus consisted of a gray cylindrical arena, 82 cm in diameter with 30 cm high aluminum walls. The arena floor was divided into three concentric circles, demarcated by black paint. The smallest inner circle had a diameter of 20 cm measured from the center of the arena. The second circle had a diameter of 50 cm and the arena wall defined the outer limit of the third circle. Each of the outer circles was divided by radial lines into equally sized areas of approximately 251 cm^2^. A light (100 W) was located 150 cm directly above the center of the open field. Performance in the open-field was scored based on latency to leave the center segment of the open-field and number of line crossings (segments entered by all four limbs) made during a 2 min time window. The open field was wiped with a soap solution between the testing of each rat.

### Acoustic startle reactivity

Twenty-four hours after the open-field test, all rats were evaluated for sensorimotor reactivity in the acoustic startle reactivity test (previously described in Servatius et al., 1998). Rats were placed on platform accelerometers (Coulbourn Instruments, Langhorne, PA) in restrainers and allowed to acclimate to the testing apparatus for 10 min prior to the onset of testing. Each 40 min testing session consisted of 60 trials of exposure to a single white noise burst (102 dB, 100 ms) against a continuous ambient background noise level of 68 dB. The inter-stimulus interval varied between 25 and 35 s. The test chambers were wiped with a soap solution between the testing of each rat. The test was performed with the ventilation fans on and the lights off. A startle response was scored if the activity exceeded a response threshold amplitude during a 250 ms baseline window prior to the onset of the stimulus. Threshold was defined as activity that exceeded 6X the standard deviation of the baseline activity. If movement did not exceed the threshold, no startle response was scored for that trial. Startle magnitude was calculated by correcting the response amplitude by body weight of the rat. Data was analyzed as mean startle magnitude of 60 consecutive trials.

### Lever-press escape/avoidance (E/A) training

Training was conducted in 16 identical operant chambers (Coulbourn Instruments, Langhorn, PA), enclosed in sound attenuated boxes (previously described in Servatius et al., 1998). Foot-shocks (1.0 mA) were delivered through a grid floor (Coulbourn Instruments, Langhorn, PA). The auditory WS was a 1000 Hz, 75 dB tone, against a 10 dB background noise. A 3 min inter-trial interval (ITI) was identified with a 5 Hz flashing light located above the lever. Graphic State Notation software (v. 3.02, Coulbourn Instruments, Langhorn, PA) controlled the stimuli and recorded response times.

Each session began with a 60 s stimulus free period, which was followed by the presentation of the WS (60 s maximum). A lever-press during the WS was considered an *avoidance response* and prevented shock exposure for both training conditions. In the contingent WS protocol, the avoidance response immediately terminated the WS and initiated the ITI. In the non-contingent WS protocol, the WS remained on for the full 60 s regardless of an avoidance response. A maximum of 99 foot-shocks (0.5 s in duration) could be delivered in the absence of an avoidance response with an inter-shock-interval of 3 s. A lever-press during the shock delivery was considered an *escape response* and immediately terminated the shock train and initiated the ITI for both training conditions. Each training session consisted of 20 trials.

### Estrous cycle

Estrous cycle of all female SD and WKY rats was determined through vaginal smears in order to explore the possible effects of avoidance learning on estrous cycling. Vaginal smears occurred 24 h after each training session, on days that E/A training did not occur. To be able to account for a pre-training difference in the cycling patterns of SD and WKY rats, 3 additional data points (each separated by 24 h) were obtained prior to E/A training. The smear procedure consisted of sampling the cells of the vaginal canal with sterile saline using a glass pipette. The recovered solution was placed on microscope slides, stained with cresyl violet, and dried. Dried slides were histologically examined under a medium power microscope (Leica, 20×/0.70 of magnification). Each slide was classified as being in the proestrus, estrus, or diestrus phase of the estrous cycle as described by Sharp and La Regina (1998).

Taking the length of each estrous phase into account, each session block of the training was assigned numerical values (“1” = if a rat was in a different estrous phase in each data point of a given session block, “2” = if a rat was in the same estrous phase for two consecutive data points of a given session block, “3” = if a rat was in the same estrous phase for all 3 data points of a given session block). Mean and SEM values of both strains were determined for each session block. Higher values indicated stagnancy and therefore greater irregularity in estrous cycle. Pre-training values of the estrous cycle were included as a covariate factor in the analysis.

### Testing schedule

Prior to E/A training, SD and WKY rats were evaluated in the open-field test and then for sensory reactivity in the acoustic startle test. Rats from each strain were stratified based on the magnitude of their startle response and then randomly assigned within each stratum to either “Contingent WS” or “Non-contingent WS” training protocols for E/A training. A total of 15 E/A training sessions occurred 3 times per week (every 2–3 days). Rats that failed to emit a single lever-press response by the end of the 4th session were omitted from the study (local IACUC standard). Two SD and two WKY rats were omitted from the study for this reason.

### Data analysis

The number and the latency of all lever-press responses as well as the number of shocks delivered were collected by Graphic State (Coulbourn Instruments, Langhorn, PA). Data were subsequently processed and collated using S-Plus (InsightfulCorp). All data were expressed as means ± standard error of the mean (SEM). Statistical results were reported only where significant differences were found. Data from the open field and acoustic startle reactivity test were analyzed using a *t*-test for independent groups. For avoidance acquisition, mean values were obtained for each of five session blocks consisting of 3 consecutive training sessions per block. Acquisition of avoidance responses was analyzed as percent avoidance (percentage of trials per session block for which an avoidance response was emitted). Avoidance latencies were naturally skewed to the onset of the WS, especially with the WS termination contingency. Therefore, all latencies were log transformed prior to analysis.

## Results

### Open-field test

WKY rats exhibited less activity compared to SD rats in the open field test. WKY rats exhibited longer latencies (12.59 ± 1.36 s) to leave the center of the open-field compared to SD rats (7.061 ± 0.57 s), *t*_(93)_ = −3.822, *p* < 0.001. Moreover, WKY rats exhibited reduced activity (26.04 ± 2.27 segments) compared to SD rats (65.39 ± 3.78 segments), *t*_(93)_ = 8.78, *p* < 0.001. Furthermore, females exhibited greater activity (32.73 ± 3.03 segments) compared to males (60.23 ± 30.34 segments), *t*_(93)_ = −5.15 (*p* < 0.05).

### Acoustic startle reactivity

WKY rats had greater magnitudes of acoustic startle responses (3.90 ± 0.24 AU) compared to SD rats (2.12 ± 0.1 AU), *t*_(93)_ = −7.15 (*p* < 0.001).

### Avoidance acquisition

**Figure 1:**
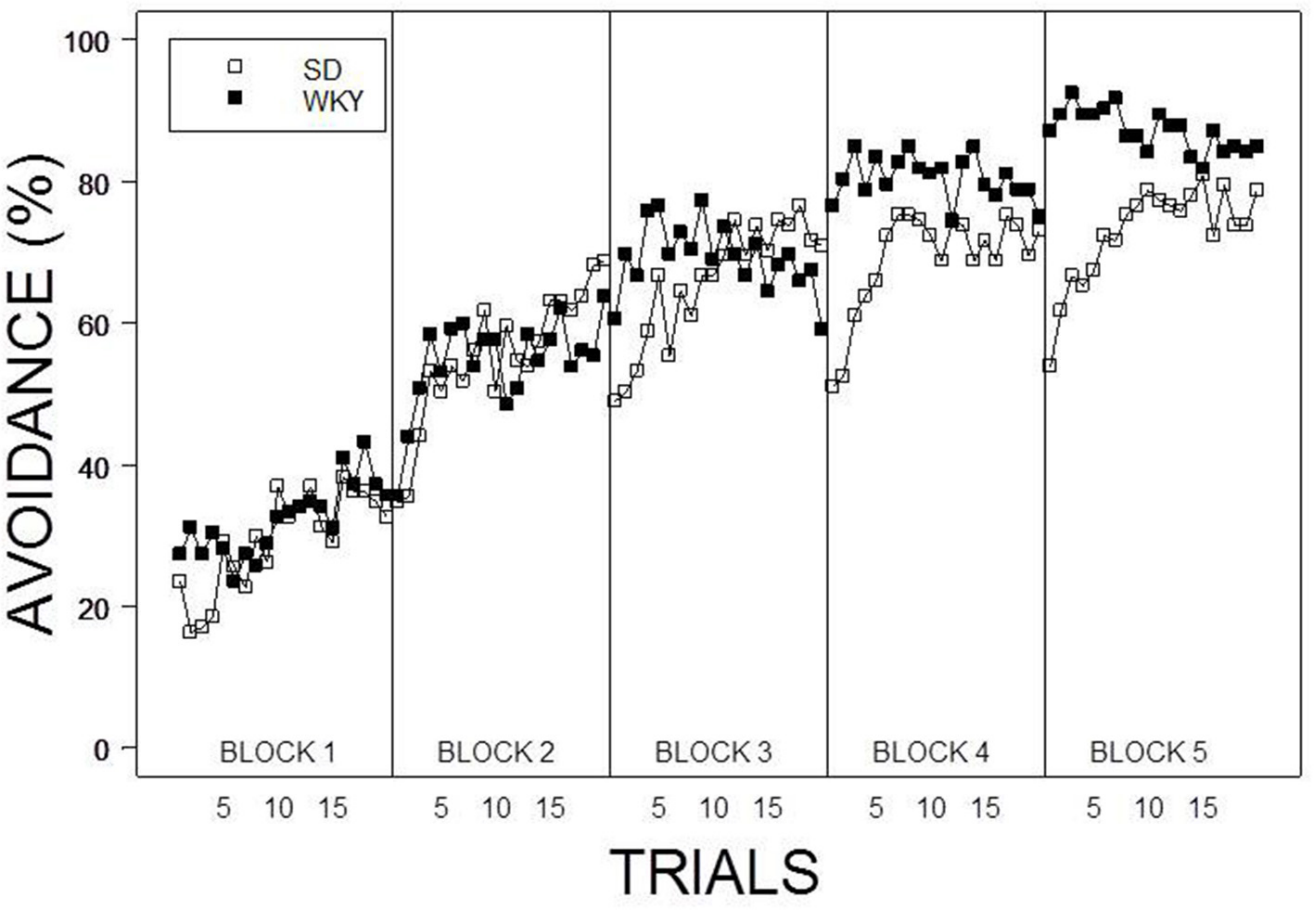
**Average percentage of avoidance responses across the 20 trials within each session block**. WKY rats acquired lever-press responses faster and to a greater asymptotic degree compared to SD rats regardless of Sex and Training. Furthermore, a lack of warm up effect was evident in within session performance of WKY rats. Each data point represents group mean ± s.e.m. (SD; *n* = 47, WKY; *n* = 44).

**Figure 2:**
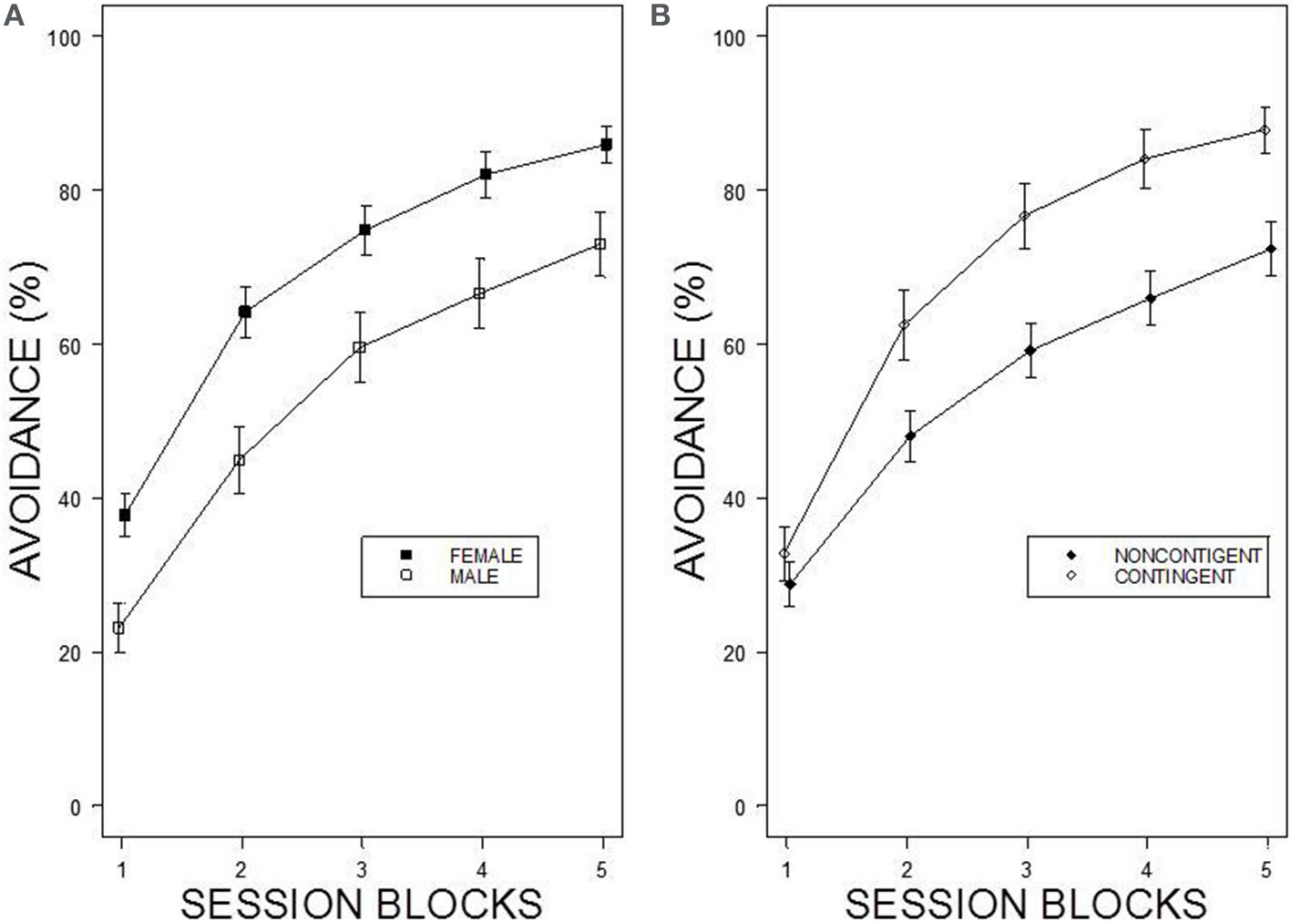
**Percentage of avoidance responses across the 5 blocks of the training**. **(A)** The left panel shows sex differences in avoidance acquisition and expression. Female rats exhibit facilitated avoidance acquisition regardless of Strain and Training. Each data point represents group mean ± s.e.m. (Female; *n* = 47, Male; *n* = 44). **(B)** The right panel shows the difference in performance between two training protocols. Rats acquired avoidance significantly faster and to a higher degree under contingent WS protocol compared to non-contingent WS protocol. Each data point represents group mean ± s.e.m. (Contingent; *n* = 43, Non-contingent; *n* = 48).

Under the contingent WS protocol, all rats acquired avoidance incrementally over training, with an asymptotic performance at least ~80%. Overall, female rats showed superior avoidance acquisition and expression compared to male rats (**Figure 2A**). Moreover, WKY rats compared to SD rats acquired avoidance responses faster and to a greater asymptotic level regardless of the training parameters (Figure 1). Consistent with previous data, strain differences were also observed in within session performance (Servatius et al., 2008; Beck et al., 2010, 2011; Jiao et al., 2011). Strain differences in within session avoidance performance were most notable in session block 4 and 5; especially in the early trials of those session blocks (Figure 1). In the non-contingent WS protocol, a decrement in avoidance performance was observed for all groups compared to the contingent WS protocol (Figure 2B). However, the slowest group (male SD rats) still attained a 50% avoidance rate by the last session block of the training. These impressions were confirmed by a 2 × 2 × 2 × 20 × 5 (Strain × Sex × Training × Trial × Session Block) mixed design analysis of variance (ANOVA). The main effect of Sex [*F*_(1, 83)_ = 14.98], as well as the Training × Session Block interaction [*F*_(4, 332)_ = 3.77] and a triple interaction of Strain × Trial × Session Block [*F*_(76, 6308)_ = 1.29] were all significant (*p*s < 0.05). The higher level of avoidance responding in females compared to males in the first session block could be due to faster learning by the females or greater general activity, leading to increased lever pressing. Therefore, an additional analysis detailed sex differences in the acquisition and expression of avoidance responses on the first training session. In the first training session, the rate of avoidance responding was comparable between female (24 ± 3%) and male rats (16 ± 3%), [*t*_(41)_ = 1.93, *p* = 0.06]. This further statistical analysis provides evidence that females learned avoidance responses faster than male rats.

Early in training, contingent and non-contingent WS protocols revealed similar avoidance latencies. However, only the contingent WS group exhibited reduced latencies to avoid across E/A training, while the non-contingent WS group continued to exhibit relatively unchanging avoidance latencies throughout the training. As a result, in the last session block of the training, non-contingent WS group had greater avoidance latencies (18.65 ± 0.38 s) compared to contingent WS group (12.27 ± 0.29 s). Furthermore, males had greater latencies (16.70 ± 0.41 s) compared to females (14.52 ± 0.30 s). A 2 × 2 × 2 × 5 (Strain × Sex × Training × Session Block) mixed-ANOVA confirmed these impressions. The test revealed a main effect of Training, *F*_(1, 83)_ = 32.95 as well as a main effect of Sex *F*_(1, 83)_ = 4.25 (*ps < 0.05)*.

### Estrous cycle

For both strains, avoidance training significantly disrupted regular cycling patterns that were observed prior to training. The highest rate of irregularity was evident during the 2nd and the 3rd session block of avoidance training both for SD and WKY rats (Table 1). However, WKY rats appeared to exhibit a faster recovery of their estrous cycle compared to SD rats. A 2 × 4 (Strain × Session Block) mixed-ANOVA confirmed these impressions as an interaction of Session Block × Strain was evident [*F*_(1, 176)_ = 3.77; *p* < 0.05].

**Table 1:** **The effect of avoidance acquisition on the estrous cycle over the 5 session blocks of the training**.

	**Pre-E/A**	**Block 1**	**Block 2**	**Block 3**	**Block 4**	**Block 5**
SD	1.33 ± 0.10	1.58 ± 0.13	1.96 ± 0.13	2.17 ± 0.14	2.13 ± 0.14	1.21 ± 0.08
WKY	1.13 ± 0.07	1.74 ± 0.11	2.0 ± 0.14	1.83 ± 0.12	1.52 ± 0.10	1.30 ± 0.10

*Irregular cycling was evident for both SD and WKY rats starting from block 1. Moreover, both strains showed recovery of the estrous cycle to a similar degree by the end of block 5. Yet, WKY rats exhibited a faster recovery of their estrous cycle compared to SD rats*.

## Discussion

Discrete trial lever press avoidance has had a long, but intermittent history. Lever press avoidance is acquired slowly compared to other types of responses; a virtue from the stand point of specificity, a bane in terms of throughput. However, relatively rapid avoidance acquisition was demonstrated by the work of Berger and Brush (1975) with the introduction of a set of procedures recapitulated decades later. Among the procedures was using a protocol in which the WS co-terminated with the shock. Another modification was having multiple sensory modalities contingent upon the requisite response (lack of the presence of shock with the passage of time, termination of the WS, and initiation of a flashing light as explicit safety). These multiple lines of reinforcement may have constituted a successful solution to the behavioral problem of observing acquisition through auto-shaping of the lever-press response. However, multiple reinforcement contingencies obscure the source of reinforcement and hinder efforts to understand neurobiological underpinnings. Which motivation drives rats to lever press? Is it escape from the WS? Prevention of impending shock? Or experiencing the safety signal?

Although experiments in shuttle box and jump up avoidance have addressed many of these concerns, an explicit test was warranted. Herein, rats were trained without reinforcement in the form of WS termination. Similar to results obtained with shuttle box (Sidman, 1955; Kamin, 1956; Sidman and Boren, 1957; Keehn, 1959; Lockard, 1963; Bower et al., 1965; Bolles et al., 1966; Bolles and Grossen, 1969), avoidance responses were acquired when lever presses were not associated with WS termination. Clearly, these data rule out one possible confounding explanation of acquisition of the lever-press avoidance, that is, escape from the WS. However, that is not to say that WS termination did not have reinforcement value. This was most evident in male SD rats, which exhibited the largest performance differential between the two contingency protocols. The reinforcement value was especially evident in terms of avoidance latencies, which were considerably longer in the non-contingent WS protocol. These data are generally in line with previous work, WS durations in other protocols varied from 5 to 10 s. Even in a trace procedure the WS was 2 s and the trace interval was 8 s (Bolles and Grossen, 1969). With avoidance latencies in the non-contingent WS protocol generally between 5 and 25 s in the final session block of training, the delay between avoidance responding and WS termination was considerably longer. Thus, the contrast between the contingent and non-contingent conditions was far greater herein.

In addition to the effects on avoidance acquisition, contingent and non-contingent WS could also affect other aspects of avoidance, such as extinction. The persistence of avoidance responses is clearly an important issue and a contributing factor in the difficulty in treating anxiety disorders. Moreover, WKY rats display persistent avoidance responding as compared to SD rats, and this persistence is dependent on shock intensity (Jiao et al., 2011). With regard to contingent and non-contingent WS protocols, extinction of avoidance following the contingent protocol might be expected to be slower than following the non-contingent protocol because avoidance was learned to a greater degree in the contingent protocol. Alternatively, delayed feedback in the non-contingent protocol could serve to slow the extinction process. It will be important to compare extinction in contingent and non-contingent protocols for SD and WKY rats in future studies.

With an emphasis on diathesis as expressed in avoidance acquisition, two vulnerabilities were studied: female sex and strain differences in temperament. Analysis showed that these factors were independent sources of vulnerability, so they will be separately discussed.

Consistent with the literature, females acquired avoidance faster and to a higher degree than male rats. This is most apparent in SD rats. Moreover, females are generally quicker to respond than male rats, regardless of the WS contingency. Beyond sex differences, we compared strain differences in learning within female rats. To our knowledge, this is the first report detailing the effect of avoidance acquisition on the estrus cycle. Exposure to avoidance context is stressful and hence, can disrupt regular cycling. Irregular cycling was evident in female rats early in training, however over the course of training—which covers numerous cycles—estrus returned to normal in almost all rats. The rate of recovery did not reflect the different training parameters. That is, in female rats the degree of difference between contingent and non-contingent WS termination was minimal. A similar pattern was observed in WKY females, albeit WKY females normalized faster. Although it is tempting to relate the faster normalization to overall better avoidance performance in female WKY rats, female WKY rats generally received more shock than female SD rats. This was apparent early in training (the first two session blocks of the training), with the two strains experiencing shock during the latter session blocks essentially at the same rate. Therefore, our data suggested that the reduction of stress (recovery of irregular cycling) as avoidance response is acquired and reliably performed is not indicative of continued stress in pathological avoidance, represented by WKY rats. The relationship between avoidance acquisition and estrus cycle would be clearer with appropriate yoked controls for number, density, control, and prediction. Nonetheless, the process of physiological adaptation to stress in females confronted with avoidance learning is an intriguing finding.

Consistent with previous demonstrations (Servatius et al., 2008; Beck et al., 2010, 2011; Jiao et al., 2011), WKY rats acquired and expressed avoidance to a higher degree than SD rats; the strains exhibited substantially different patterns of avoidance responding that were evident in both between and within session performance. One recurring pattern in within session performance is that in contrast to SD rats, WKY rats exhibit avoidance on the first trial of each session. This first trial avoidance is expressed soon after avoidance responses become numerous and insulates WKY rats to experience changes in response contingency. As a result, avoidance responding perseverates throughout the process of extinction—even to the extent that avoidance performance is 5%, that is, only the first trial of a session (Servatius et al., 2008; Beck et al., 2011; Jiao et al., 2011). Initial avoidance, once established, is apparent in feral rats, those with septal lesions, or when shocks are delivered prior to the first WS, leading to the conclusion that first trial avoidance is secondary to differences in arousal. The first trial avoidance of WKY rats does not seem to fit this characterization. Inspection of the avoidance latency data shows avoidance latencies are similar between the first trial and subsequent trials. This similarity is also evident in WKY rats trained under non-contingent WS protocol. These longer latencies in the first trial of those trained with a termination contingency and those without suggests that first trial avoidance is considered, not evoked by the contextual elements, which are the same in both groups. The robustness of first trial avoidance in WKY rat provides a potential therapeutically-relevant target for pharmacological manipulations.

Discrete-trial lever press avoidance can inform us about the development of both adaptive and maladaptive coping strategies. Stripped of reinforcement from WS termination, it is clear that avoidance reinforcement is from future events, whether through absence of shock or the inherent conception of safety. With expectations come decisions on whether to respond or not, knowing failure could result in the experience of foot shock. Outbred rats temper the avoidance with escape; escape providing actual evidence of shock presence. Willingness to experience the shock allows for increased sensitivity to changes in shock presence, thereby allowing for and facilitating extinction. Female SD rats, although expressing avoidance to a higher degree than male rats, do not express avoidance to the degree that they are insulated from the presence or absence of shock. Thus, sex differences are confined to rates of acquisition and expression.

The expectations of such aversive future events does not depend on the amygdala (unpublished observations). The essential neural circuitry for normal acquisition of avoidance expectancies awaits further research. Inbred WKY rats are more driven by the expectation of shock, expressing avoidance from the first trial of a session. This motivation may contribute to the facilitation of associative learning evident in classical eyeblink conditioning and the lack of latent inhibition by WKY rats (Ricart et al., 2011a), or it may be additional to such enhanced associative processes. Facilitation of avoidance expressed by WKY rats appears to depend on the amygdala (unpublished observations). This distinction echoes amygdala differences observed in those with anxiety disorders compared to otherwise healthy individuals (Liberzon et al., 1999; Shin et al., 2004).

In summary, sex and strain differences are apparent in avoidance acquisition and expression regardless of a WS termination contingency, indicating enhanced sensitivity for the expectation of future aversive events. In contrast to simple non-associative and associative processes, the neurobiology of avoidance and expectancy is not fully defined. This finding has the promise of providing critical insights into the neurobiology of extreme avoidance expression in anxiety disorders, but also other stress related pathology in which maladaptive coping is a common feature.

### Conflict of interest statement

The authors declare that the research was conducted in the absence of any commercial or financial relationships that could be construed as a potential conflict of interest.

## References

[B1] American Psychiatric Association. (2000). Diagnostic and Statistical Manual of Mental Disorders, 4th-TR edn. Washington, DC: American Psychiatric Association

[B2] Beck K. D. Jiao X. Pang K. C. Servatius R. J. (2010). Vulnerability factors in anxiety determined through differences in active-avoidance behavior. Prog. Neuropsychopharmacol. Biol. Psychiatry 34, 852–860 10.1016/j.pnpbp.2010.03.036 20382195

[B3] Beck K. D. Jiao X. Ricart T. M. Myers C. E. Minor T. R. Pang K. C. (2011). Vulnerability factors in anxiety: strain and sex differences in the use of signals associated with non-threat during the acquisition and extinction of active-avoidance behavior. Prog. Neuropsychopharmacol. Biol. Psychiatry 35, 1659–1670 10.1016/j.pnpbp.2011.05.002 21601608

[B4] Beck K. D. McLaughlin J. Bergen M. T. Cominski T. P. Moldow R. L. Servatius R. J. (2008). Facilitated acquisition of the classically conditioned eyeblink response in women taking oral contraceptives. Behav. Pharmacol. 19, 821–828 10.1097/FBP.0b013e32831c3b82 19020417

[B5] Beck K. D. Wasserman M. C. Furst S. J. Pang K. C. Servatius R. J. (2012). Differential effects of progesterone and medroxyprogesterone on delay eyeblink conditioning in ovariectomized rats. Neurobiol. Learn. Mem. 97, 148–155 10.1016/j.nlm.2011.11.002 22138327

[B68] Berger D. F. Brush F. R. (1975). Rapid acquisition of discrete-trial lever-press avoidance: effects of signal-shock interval. J. Exp. Anal. Behav. 24, 227–239 10.1901/jeab.1975.24-227 16811875 PMC1333403

[B6] Berger D. F. Starzec J. J. Mason E. B. (1981). The relationship between plasma corticosterone levels and lever-press avoidance vs. escape behaviors in rats. Physiol. Psychol. 9, 81–86 10.3758/BF03326962

[B7] Bersh P. J. Alloy L. B. (1978). Avoidance based on shock intensity reduction with no change in shock probability. J. Exp. Anal. Behav. 30, 293–300 10.1901/jeab.1978.30-293 16812109 PMC1332773

[B8] Bleich A. Gelkopf M. Melamed Y. Solomon Z. (2006). Mental health and resiliency following 44 months of terrorism: a survey of an Israeli national representative sample. BMC Med. 4:21 10.1186/1741-7015-4-21 16934160 PMC1560155

[B9] Bolles R. C. (1970). Species-specific defense reactions and avoidance learning. Psychol. Rev. 77, 32–48 10.1037/h0028589

[B10] Bolles R. C. Grossen N. E. (1969). Effects of an informational stimulus on the acquisition of avoidance behavior in rats. J. Comp. Physiol. Psychol. 68, 90–99 10.1037/h0027677

[B11] Bolles R. C. Stokes L. Younger M. S. (1966). Does CS termination reinforce behavior? J. Comp. Physiol. Psychol. 62, 201–207 10.1037/h0023678 5969598

[B12] Bower G. Starr R. Lazarovitz L. (1965). Amount of response-produced change in the CS and avoidance learning. J. Comp. Physiol. Psychol. 59, 13–17 10.1037/h0021623 14282395

[B13] Braw Y. Malkesman O. Dagan M. Bercovich A. Lavi-Avnon Y. Schroeder M. (2006). Anxiety-like behaviors in pre-pubertal rats of the Flinders Sensitive Line (FSL) and Wistar-Kyoto (WKY) animal models of depression. Behav. Brain Res. 167, 261–269 10.1016/j.bbr.2005.09.013 16271773

[B15] Coover G. D. Ursin H. Levine S. (1973). Plasma corticosterone levels during active avoidance learning in rats. J. Comp. Physiol. Psychol. 82, 170–174 4684973 10.1037/h0033790

[B17] Declercq M. DeHouwer J. (2008). On the role of US expectancies in avoidance behavior. Psychol. Bull. Rev. 15, 99–102 10.3758/PBR.15.1.99 18605487

[B18] Dymond S. Roche B. (2009). A contemporary behavior analysis of anxiety and avoidance. Behav. Anal. 32, 7–27 22478511 10.1007/BF03392173 PMC2686994

[B19] Ferguson S. A. Cada A. M. (2004). Spatial learning/memory and social and nonsocial behaviors in the spontaneously hypertensive, Wistar-Kyoto and Sprague-Dawley rat strains. Pharmacol. Biochem. Behav. 77, 583–594 10.1016/j.pbb.2003.12.014 15006470

[B20] Foa E. B. Stein D. J. McFarlane A. C. (2006). Symptomatology and psychopathology of mental health problems after disaster. J. Clin. Psychiatry 67, 15–25 16602811

[B21] Fox N. A. Henderson H. A. Marshall P. J. Nichols K. E. Ghera M. M. (2005). Behavioral inhibition: linking biology and behavior within developmental framework. Annu. Rev. Psychol. 56, 235–262 10.1146/annurev.psych.55.090902.141532 15709935

[B22] Genazzani A. R. Petraglia F. De Ramundo B. M. Genazzani A. D. Amato F. Algeri I. (1991). Neuroendocrine correlates of stress-related amenorrhea. Ann. N.Y. Acad. Sci. 626, 125–129 10.1111/j.1749-6632.1991.tb37906.x 2058948

[B23] Gonzalez A. S. Rodriguez Echandla E. L. Cabrera R. Foscolo M. R. (1994). Neonatal chronic stress induces subsensitivity to chronic stress in adult rats: II. Effects on estrous cycle in females. Physiol. Behav. 56, 591–595 10.1016/0031-9384(94)90306-9 7972413

[B24] Hapke U. Schumann A. Rumpf H. J. John U. Meyer C. (2006). Post-traumatic stress disorder: the role of trauma, pre-existing psychiatric disorders, and gender. Eur. Arch. Psychiatry Clin. Neurosci. 256, 299–306 10.1007/s00406-006-0654-6 16783498

[B25] Heinsbroek R. P. van Oyen H. G. van de Poll N. E. Boer G. J. (1983). Failure of dexamethasone to influence sex differences in acquisition of discriminated lever press avoidance. Pharmacol. Biochem. Behav. 19, 599–604 10.1016/0091-3057(83)90334-9 6647498

[B26] Hineline P. N. (2001). Beyond the molar-molecular distinction: we need multiscaled analyses. J. Exp. Anal. Behav. 75, 342–347 10.1901/jeab.2001.75-342 11453624 PMC1284823

[B27] De Houwer J. Crombez G. Baeyens F. (2005). Avoidance behavior can function as a negative occasion setter. J. Exp. Psychol. Anim. Behav. Process. 31, 101–106 10.1037/0097-7403.31.1.101 15656731

[B28] Jiao X. Pang K. C. Beck K. D. Minor T. R. Servatius R. J. (2011). Avoidance perseveration during extinction training in Wistar-Kyoto rats: an interaction of innate vulnerability and stressor intensity. Behav. Brain Res. 221, 98–107 10.1016/j.bbr.2011.02.029 21376086 PMC3079807

[B29] Kamin L. J. (1956). The effects of termination of the CS and avoidance of the US on avoidance learning. J. Comp. Physiol. Psychol. 49, 420–424 10.1037/h0088011 13345924

[B31] Karamustafalioglu O. K. Zohar J. Guveli M. Gal G. Bakim B. Fostick L. (2006). Natural course of posttraumatic stress disorder: a 20-month prospective study of Turkish earthquake survivors. J. Clin. Psychiatry 67, 882–889 10.4088/JCP.v67n0604 16848647

[B32] Keehn J. D. (1959). The effect of a warning signal on unrestricted avoidance behavior. Br. J. Psychol. 50, 125–135 10.1111/j.2044-8295.1959.tb00690.x

[B70] Liberzon I. Taylor S. F. Amdur R. Jung T. D. Chamberlain K. R. Minoshima S. (1999). Brain activation in PTSD in response to trauma-related stimuli. Biol. Psychiatry 45, 817–826 10202568 10.1016/s0006-3223(98)00246-7

[B33] Lockard J. S. (1963). Choice of a warning signal or no warning signal in an unavoidable shock situation. J. Comp. Physiol. Psychol. 56, 526–530 10.1037/h0041552 13931179

[B34] Lovibond P. F. (2006). An integrated expectancy model, in Fear and Learning: From Basic Processes to Clinical Implications, eds Craske M. G. Hermans D. Vansteenwegen D. (Washington, DC: American Psychological Association), 117–132

[B37] McAuley J. D. Stewart A. L. Webber E. S. Cromwell H. C. Servatius R. J. Pang K. C. (2009). Wistar-Kyoto rats as an animal model of anxiety vulnerability: support for a hypervigilance hypothesis. Behav. Brain Res. 204, 162–168 10.1016/j.bbr.2009.05.036 19523988 PMC2723189

[B38] McCarty R. Kirby R. F. Garn P. G. (1984). Strain differences in sympathetic-adrenal medullary responsiveness and behavior. Behav. Neural Biol. 40, 98–113 10.1016/S0163-1047(84)90206-1 6732708

[B40] Mitchell C. J. De Houwer J. Lovibond P. F. (2009). The propositional nature of human associative learning. Behav. Brain Sci. 32, 183–198 10.1017/S0140525X09000855 19386174

[B42] Mowrer O. H. (1956). Two-factor learning theory reconsidered, with special reference to secondary reinforcement and the concept of habit. Psychol. Rev. 63, 114–128 10.1037/h0040613 13310707

[B43] Norman R. L. McGlone J. Smith C. J. (1994). Restraint inhibits luteinizing hormone secretion in the follicular phase of the estrous cycle in rhesus macaques. Biol. Reprod. 50, 16–26 10.1095/biolreprod50.1.16 8312440

[B45] Owen J. W. Herdegen R. T. Cicala G. A. (1977). Warning signal termination does not function as a feedback signal. Bull. Psychon. Soc. 10, 295–297 10.3758/BF03329340

[B46] Pare W. P. (1989). Stress ulcer and open-field behavior of spontaneously hypertensive, normotensive, and Wistar rats. Pavlov. J. Biol. Sci. 24, 54–57 2726299 10.1007/BF02964537

[B47] Pare W. P. Schimmel G. T. (1986). Stress ulcer in normotensive and spontaneously hypertensive rats. Physiol. Behav. 36, 699–705 10.1016/0031-9384(86)90357-4 3714844

[B48] Perrotti L. I. Dennis T. S. Jiao X. Servatius R. J. Pang K. C. Beck K. D. (2013). Activation of extracellular signal-regulated kinase (ERK) and Delta FosB in emotion-associated neural circuitry after asymptotic levels of active avoidance behavior are attained. Brain Res. Bull. 98, 102–110 10.1016/j.brainresbull.2013.07.004 23932962

[B49] Ricart T. M. De Niear M. A. Jiao X. Pang K. C. Beck K. D. Servatius R. J. (2011a). Deficient proactive interference of eyeblink conditioning in Wistar-Kyoto rats. Behav. Brain Res. 216, 59–65 10.1016/j.bbr.2010.07.005 20621128 PMC2975831

[B50] Ricart T. M. Jiao X. Pang K. C. Beck K. D. Servatius R. J. (2011b). Classical and instrumental conditioning of eyeblink responses in Wistar-Kyoto and Sprague-Dawley rats. Behav. Brain Res. 216, 414–418 10.1016/j.bbr.2010.08.029 20801161 PMC3057661

[B51] Rosenbaum J. F. Biederman J. Bolduc-Murphy E. A. Faraone S. V. Chaloff J. Hirshfeld D. R. (1993). Behavioral inhibition in childhood: a risk factor for anxiety disorders. Harv. Rev. Psychiatry 1, 2–16 10.3109/10673229309017052 9384823

[B52] Rosenbaum J. F. Biederman J. Hirshfeld D. R. Bolduc E. A. Faraone S. V. Kagan J. (1991). Further evidence of an association between behavioral inhibition and anxiety disorders: results from a family study of children from a non-clinical sample. J. Psychiatr. Res. 25, 49–65 10.1016/0022-3956(91)90015-3 2027095

[B53] Schneier F. R. (2003). Social anxiety disorder: is common, underdiagnosed, impairing, and treatable. BMJ 327, 515–516 10.1136/bmj.327.7414.515 12958087 PMC192835

[B54] Seligman M. E. Johnston J. C. (1973). A Cognitive Theory of Avoidance Learning. Oxford: VH Winston & Sons

[B55] Servatius R. J. Jiao X. Beck K. D. Pang K. C. Minor T. R. (2008). Rapid avoidance acquisition in Wistar-Kyoto rats. Behav. Brain Res. 192, 191–197 10.1016/j.bbr.2008.04.006 18501974

[B56] Servatius R. J. Ottenweller J. E. Beldowicz D. Guo W. Zhu G. Natelson B. H. (1998). Persistently exaggerated startle responses in rats treated with pyridostigmine bromide. J. Pharmachol. Exp. Ther. 287, 1020–1028 9864288

[B57] Servatius R. J. Ottenweller J. E. Natalson B. H. (1995). Delayed startle sensitization distinguishes rats exposed to one or three stress session: further evidence toward an animal model of PTSD. Biol. Psychiatry 38, 539–546 10.1016/0006-3223(94)00369-E 8562666

[B58] Sharp P. E. La Regina M. C. (1998). The Laboratory Rat. Boca Raton, FL: CRC Press

[B69] Shin L. M. Orr S. P. Carson M. A. Rauch S. L. Macklin M. L. Lasko N. B. (2004). Regional cerebral blood flow in amygdala and medial prefrontal cortex during traumatic imagery in male and female Vietnam veterans with PTSD. Arch. Gen. Psychiatry 61, 168–176 10.1001/archpsyc.61.2.168 14757593

[B59] Shors T. J. Ary J. P. Eriksen K. J. Wright K. W. (1986). P100 amplitude variability of the pattern visual evoked potential. Electroencephalogr. Clin. Neurophysiol. 65, 316–319 10.1016/0168-5597(86)90010-9 2424744

[B60] Shors T. J. Lewczyk C. Pacynski M. Mathew P. R. Pickett J. (1998). Stages of estrous mediate the stress-induced impairment of associative learning in the female rat. Neuroreport 9, 419–423 10.1097/00001756-199802160-00012 9512383

[B61] Sidman M. (1955). Some properties of the warning stimulus in avoidance situation. J. Comp. Physiol. Psychol. 48, 444–450 10.1037/h0047481 13271615

[B62] Sidman M. Boren J. J. (1957). A comparison of two types of warning stimulus in an avoidance situation. J. Comp. Physiol. Psychol. 50, 282–287 10.1037/h0046474 13463166

[B63] Smith R. P. Larkin G. L. Southwick S. M. (2008). Trends in U.S. emergency department visits for anxiety-related mental health conditions, 1992–2001. J. Clin. Psychiatry 69, 286–94 10.4088/JCP.v69n0215 18363455

[B65] Vogt D. S. Pless A. P. King L. A. King D. W. (2005). Deployment stressors, gender, and mental health outcomes among Gulf War I veterans. J. Trauma Stress 18, 272–284 10.1002/jts.20018 16281224

[B66] Wittchen H. U. Hoyer J. (2001). Generalized anxiety disorder: nature and course. J. Clin. Psychiatry 62, 15–19 11414546

[B67] Wood G. E. Shors T. J. (1998). Stress facilitates classical conditioning in males, but impairs classical conditioning in females through activational effects of ovarian hormones. Proc. Natl. Acad. Sci. U.S.A. 95, 4066–4071 10.1073/pnas.95.7.4066 9520494 PMC19964

